# Risk Factors for Pathologically Confirmed Lymph Nodes Metastasis in Patients With Clinical T2N0M0 Stage Prostate Cancer

**DOI:** 10.3389/fonc.2020.01547

**Published:** 2020-08-14

**Authors:** Ning Xu, Zhi-Bin Ke, Ye-Hui Chen, Yu-Peng Wu, Shao-Hao Chen, Yong Wei, Qing-Shui Zheng, Jin-Bei Huang, Xiao-Dong Li, Xue-Yi Xue

**Affiliations:** Department of Urology, The First Affiliated Hospital of Fujian Medical University, Fuzhou, China

**Keywords:** PI-RADS, tumor burden, Gleason grading group, lymph node metastasis, prostate cancer

## Abstract

**Objective:**

To explore the risk factors for postoperatively pathological lymph node metastasis in patients with clinical T2N0M0 stage prostate cancer (PCa).

**Methods:**

We retrospectively analyzed clinicopathological data of 316 patients with clinical T2 stage PCa and preoperative negative lymph nodes [LN(−)] indicated by imaging (cT2N0M0) between January 2014 and May 2019. Multivariate logistic regression analysis was performed to determine risk factors for postoperatively pathological pLN(+) in patients with cT2N0M0 stage PCa. Spearman correlation analysis was used to explore the relationship between tumor burden and Prostate Imaging Reporting and Data System version 2 (PI-RADS v2) score.

**Results:**

A total of 45 patients (14.2%) were confirmed by postoperative pathology to have LN metastasis. Univariate analysis indicated that total prostate-specific antigen (tPSA), PI-RADS v2 score, postoperative Gleason grade group (GGG), intraductal carcinoma of the prostate (IDC-P), clinical T2 substaging, and postoperative pathological tumor burden were risk factors for pLN(+) in all patients. Multivariate analysis showed that tPSA and postoperative GGG were risk factors for pLN(+) in all patients. Univariate analysis revealed that tPSA, PIRADS v2 score, clinical T2 substaging, IDC-P, postoperative pathological tumor burden, and postoperative GGG were risk factors for pLN(+) in patients with GGG ≥ 3. Multivariate analysis suggested that tPSA, PI-RADS v2 score, clinical T2 substaging, postoperative pathological tumor burden, and GGG were risk factors for pLN (+) in patients with GGG ≥ 3. Spearman correlation analysis showed that PI-RADS v2 score was positively correlated with clinical T2 substaging and postoperative pathological tumor burden.

**Conclusion:**

There was a high risk of LN metastasis in patients with cT2 PCa if they had high preoperative tPSA or high postoperative GGG. Patients with cT2 PCa and GGG ≥ 3 had a high risk of LN metastasis if they had high PI-RADS v2 score, high preoperative clinical stage or high postoperative pathological tumor burden. PI-RADS v2 score predicted tumor burden well in patients with GGG ≥ 3.

## Introduction

A low prostate-specific antigen (PSA) threshold for triggering biopsy has increased the number of patients with prostate cancer (PCa) and lymph node (LN) metastasis [LN(+)] confirmed by postoperative pathology, despite being LN(−) by preoperative imaging (1). It is reported that LN metastasis frequently indicates poor prognosis and increases the probability of postoperative biochemical recurrence (2). To the best of our knowledge, LN dissection (LND) is the most direct and standard method to determine the presence of LN(+) (3). However, the incidence of LN(+) is frequently heterogeneous among patients with different Gleason scores (GS) (1). The LN metastasis rate of PCa patients with GS 8–10 is significantly increased compared with those with GS 6–7 (1, 2). Traditional GS scores and associated nomograms for assessing LN metastasis might underestimate the risk of lymphatic invasion because of old samples (4). A previous study demonstrated that LN invasion was finally detected by postoperative pathological findings in 17.9% of patients with clinical organ-localized PCa (4). Therefore, it is important for patients with intermediate- and high-risk PCa to undergo pelvic LND (PLND), which might prevent potential lymphatic invasion (5, 6). However, because the complexity of PLND significantly prolongs operating time (7), there is a tendency to increase the use of robot-assisted radical prostatectomy (RP) and decrease the use of PLND, even in patients with high-risk PCa (6). Compared with the heterogeneity of LN metastatic burden to assess prognosis (5), the pathological features of primary tumors may better predict prognosis (3, 8). Complete resection of the primary tumor might be more important than LND (3, 8). Therefore, it is crucial to explore the risk factors for LN metastasis.

Currently, the risk factors for pathologically confirmed LN metastasis in patients with clinical T2N0M0 stage PCa have not been fully elucidated. This study aimed to determine the risk factors for postoperative pathological LN metastasis in patients with preoperative negative LNs and clinical T2 stage (cT2N0M0). In particular, we explored whether the latest International Society of Urological Pathology (ISUP) Gleason Grading Group (GGG) and Prostate Imaging Reporting and Data System version 2 (PI-RADS v2) score were risk factors.

## Materials and Methods

### Patients

We included 316 patients with clinical T2 stage (cT2) PCa and negative LNs indicated by preoperative imaging (cT2N0M0) between January 2014 and May 2019 at our center. All patients underwent whole bone scanning, chest and abdominal computed tomography (CT)/multiparametric magnetic resonance imaging (mpMRI), and were diagnosed with PCa postoperatively pathologically. All patients underwent RP plus standard PLND that included the external iliac and obturator LNs (6). The clinical data and tumor pathological features were retrospectively analyzed. The exclusion criteria were as follows: (1) preoperative neoadjuvant chemotherapy/radiotherapy; (2) incomplete clinical data, unclear pathological diagnosis and lack of prostate MRI images; (3) inability to determine PI-RADS score [poor image quality; diffusion-weighted imaging (DWI)/dynamic contrast enhancement MRI functional sequence deletion; or DWI *b* value < 800 s/mm^2^]; (4) capsular infiltration or seminal vesicle metastasis suggested by preoperative imaging and confirmed by postoperative pathology; (5) positive surgical margins; and (6) other types of tumors confirmed pathologically.

### Clinical Data

Clinical data were collected, including total PSA (tPSA), clinical staging, PI-RADS v2 score, postoperative pathological Gleason score, postoperative pathological tumor burden (%), intraductal carcinoma of the prostate (IDC-P)/acinar adenocarcinoma (%) and LN status. Postoperative specimens were evaluated at our center by two pathologists with at least 10 years of experience. The 2014 ISUP GGG system was used (9). The system was divided into 5 groups (9): 1 (Gleason score ≤6), 2 (Gleason score 3 + 4 = 7), 3 (Gleason score 4 + 3 = 7), 4 (Gleason score 8), and 5 (Gleason score ≥9).

### Statistical Analysis

Statistical analysis was conducted using SPSS version 22.0 (IBM Corp., Armonk, NY, United States). Categorical data were presented as frequency (%) and analyzed by chi-square test or Fisher’s test. Continuous normally distributed data were represented as mean ± standard deviation and analyzed by *t*-test. Continuous data that were not normally distributed were analyzed by Kruskal–Wallis test. Multivariate logistic regression was performed to determine risk factors for postoperatively pathological lymph node metastasis in patients with preoperative cT2N0M0 stage PCa. Spearman correlation analysis was used to explore the correlation between PI-RADS v2 score and clinical T2 substaging and postoperative pathological tumor burden. *P* < 0.05 was considered statistically significant.

## Results

We included 316 patients with clinical T2N0M0 stage PCa, and 45 patients (14.2%) were confirmed to have LN metastasis by postoperative pathology. The number of patients with pathological LN metastasis in each grading group was as follows: 0 cases in GGG 1 (3 + 3) group; 3 cases (0.9%) in GGG2 (3 + 4) group; 9 cases (2.8%) in GGG3 (4 + 3) group; 15 cases (4.7%) in GGG4 (8) group; and 18 cases (5.7%) in GGG5 (9, 10) group. Univariate analysis indicated that tPSA, PI-RADS v2 score, postoperative GGG, intraductal carcinoma of the prostate (IDC-P), clinical T2 substaging, and postoperative pathological tumor burden were risk factors for pathological LN metastasis confirmed postoperatively in all patients (Table 1). Multivariate logistic regression analysis showed that tPSA and postoperative GGG were risk factors for pathological LN metastasis confirmed postoperatively in all patients (Table 2). Univariate analysis revealed that tPSA, PI-RADS v2 score, clinical T2 substaging, postoperative pathological tumor burden, IDC-P and postoperative GGG were risk factors for pathological LN metastasis confirmed postoperatively in patients with GGG ≥ 3 (Table 3). Multivariate analysis showed that tPSA, PI-RADS v2 score, clinical T2 substaging, postoperative pathological tumor burden, and postoperative GGG were risk factors for pathological LN metastasis confirmed postoperatively in patients with GGG ≥ 3 (Table 4). Spearman correlation analysis showed that PI-RADS v2 score was positively correlated with clinical T2 substaging and postoperative pathological tumor burden, and, the higher the grading group, the greater the correlation (Table 5).

**TABLE 1:** Comparison of clinicopathological characteristics between pLN(+) and pLN(−) in all patients.

PCa	Lymph node status		*P*-value
	**Positive**	**Negative**	
Age	67.36 ± 8.49	67.44 ± 8.76	0.955
tPSA	11.99 ± 3.49	8.45 ± 3.37	<0.001*
PI-RADS v2 score (n.)			<0.001*
1	1	30	
2	7	74	
3	11	110	
4	10	53	
5	16	4	
Clinical T2 substage (n.)			<0.001*
cT2a	8	128	
cT2b	9	93	
cT2c	28	50	
Pathological tumor burden (%)	36.77 ± 12.51	30.06 ± 13.24	0.002*
IDC-P/acinar adenocarcinoma (%)	22.89 ± 6.76	20.73 ± 6.56	0.043*
Number of LN removed (n.)			0.281
5–9	10	73	
10–14	9	80	
15–19	14	55	
≥20	12	63	
Postoperative GGG (n.)			<0.001*
1	0	27	
2	3	29	
3	9	87	
4	15	70	
5	18	58	

*P < 0.05. GGG, Gleason grading group; LN, lymph node; PI-RADS v2, Prostate Imaging Reporting and Data System version 2; tPSA, total prostate-specific antigen; IDC-P, intraductal carcinoma of the prostate.

**TABLE 2:** Multivariate logistic regression analysis of risk factors for LN invasion in all patients.

PCa	LN status (Positive/Negative)		
	P	OR	95% CI
tPSA	<0.001*	1.260	1.119–1.419
PI-RADS score	0.066	1.563	0.970–2.520
Clinical T2 substage	0.069	1.774	0.956–3.292
Pathological tumor burden (%)	0.058	1.028	0.999–1.057
IDC-P/acinar adenocarcinoma (%)	0.305	1.030	0.973–1.090
Postoperative GGG	0.030*	1.517	1.042–2.209

*P < 0.05. GGG, Gleason grading group; LN, lymph node; PI-RADS, Prostate Imaging Reporting and Data System; tPSA, total prostate-specific antigen; IDC-P, intraductal carcinoma of the prostate.

**TABLE 3:** Comparison of clinicopathological characteristics between pLN(+) and pLN(−) in patients with postoperative GGG ≥ 3.

PCa	Lymph node		*P*-value
	Positive	Negative	
tPSA	11.63 ± 3.27	8.75 ± 3.25	<0.001*
PI-RADS score (n.)			<0.001*
1	0	14	
2	5	62	
3	11	87	
4	10	48	
5	16	4	
Clinical T2 substage (n.)			<0.001*
cT2a	5	95	
cT2b	9	79	
cT2c	28	41	
Pathological tumor burden (%)	37.97 ± 12.04	29.12 ± 13.62	<0.001*
IDC-P/acinar adenocarcinoma (%)	23.19 ± 6.79	20.81 ± 6.46	0.032*
Number of LN removed (n.)			0.173
5–9	8	59	
10–14	8	60	
15–19	14	43	
≥20	12	53	
Postoperative GGG (n.)			0.039*
3	9	87	
4	15	70	
5	18	58	

*P < 0.05. GGG, Gleason grading group; LN, lymph node; PI-RADS, Prostate Imaging Reporting and Data System; tPSA, total prostate-specific antigen; IDC-P, intraductal carcinoma of the prostate.

**TABLE 4:** Multivariate logistic regression analysis of risk factors for LN invasion in patients with GGG ≥ 3.

PCa	LN (Positive/Negative)		
	P	OR	95% CI
tPSA	0.022*	1.159	1.021–1316
PI-RADS v2 score	0.033*	1.812	1.050–3.127
Clinical T2 substage	0.035*	2.104	1.052–4.205
Pathological tumor burden (%)	0.028*	1.035	1.004–1.067
IDC-P/acinar adenocarcinoma (%)	0.255	1.036	0.975–1.100
Postoperative GGG	0.029*	1.767	1.061–2.943

*P < 0.05. GGG, Gleason grading group; LN, lymph node; PI-RADS v2, Prostate Imaging Reporting and Data System version 2; tPSA, total prostate-specific antigen; IDC-P, intraductal carcinoma of the prostate.

**TABLE 5:** Correlation between tumor burden and PI-RADS v2 score.

		cT2 substage		Pathological tumor burden	
		Index	*P*	Index	*P*
PI-RADS v2	Overall	0.566	<0.001*	0.154	0.006
	GGG ≥ 3	0.659	<0.001*	0.243	<0.001*
	GGG ≥ 4	0.723	<0.001*	0.314	<0.001*
	GGG ≥ 5	0.703	<0.001*	0.371	0.001

*Spearman correlation analysis, *P* < 0.05. GGG, Gleason grading group; PI-RADS v2, Prostate Imaging Reporting and Data System version 2.

## Discussion

In 2014, ISUP hosted a consensus conference to make further revisions to the Gleason grading system of PCa. This revision not only defined the morphological criteria for each Gleason grade in more detail, but also proposed a new grading system based on prognosis, called the 2014 International Society of Urological Pathology Consensus Conference on Gleason Grading of Prostatic Carcinoma (2014 ISUP GGG system) (10). Organ-localized PCa does not metastasize to PLNs in patients with GGG ≤ 2 under the premise of negative surgical margins; therefore, there is almost no risk of progression (2, 11). Our study also revealed that patients with cT2 stage and GGG ≤ 2 PCa had a low risk of LN metastasis (0.9%). However, a previous study showed that postoperative GGG ≥ 4 predicted poor cancer-specific survival (CSS) and overall survival (OS) of patients with PCa. The predictive value of the 2014 ISUP GGG system for CSS and OS was significantly better than that of LN metastasis (3, 8). This indicates that the pathological features of the primary tumor are more effective in predicting prognosis. Consequently, complete resection of the primary tumor may be of greater importance than LND. The present study also demonstrated that poor pathological features of primary tumors indicate a higher risk of pathological LN metastasis in patients with cT2 PCa, and higher tPSA and GGG are more significantly predictive of postoperative LN metastasis.

The 8th edition of the American Joint Committee on Cancer (AJCC) system has modified the pT2 staging. The pT2 stage in the 7th edition of the AJCC system was divided into 3 substages according to the extent of tumor involvement and single/double sidedness. However, the substages did not convey prognostic information and there was no difference in prognosis between various substages (10). The relationship between clinical and pathological T stages is poor (10). Large unilateral tumors may be classified as low pT stage, whereas small bilateral tumors may be classified as high pT stage (10). Therefore, organ-localized PCa is no longer subclassified but attributed pathologically to pT2 in the 8th edition of the AJCC system although the substages (cT2a, cT2b, and cT2c) are retained in clinical T2 staging (10). However, we found that pathological LN metastasis was more likely in the cases with higher preoperative cT2 substage or greater postoperative pathological tumor burden in patients with GGG ≥ 3 and cT2 stage PCa. Therefore, whether to remove pT2 substage remains controversial. In patients with GGG ≥ 3, we might further substage pT2 according to tumor burden to guide subsequent more precise follow-up and treatments strategies, such as performing prostate-specific membrane antigen (PSMA) -PET/CT or salvage lymph node dissection (12, 13).

High PI-RADS v2 score frequently indicates high probability of clinically significant PCa, and the lesions are commonly visible and large in multiparameter MRI (mpMRI) or surgical specimens. Christopher et al. (14) reported that MR-derived tumor volume ≥2.1 cm^3^ generally predicted invasion of prostate capsule (sensitivity/specificity = 78.4/73.5%). These imaging and pathological features are inevitably associated with PCa aggressiveness or prognosis (15). In general, high PI-RADS v2 score suggests high aggressiveness and poor clinical prognosis of PCa and high risk of pLN(+) in patients with preoperative LN(−) indicated by mpMRI (16–18). However, misdiagnosis by mpMRI often occurs in patients with tumor volume <1.0 cm^3^ because these small-volume tumors are difficult to detect by mpMRI (19, 20). It is reported that patients with tumors which are not visible on MRI are frequently confirmed to have small-volume tumors and low GS after RP (0.15 vs. 1.45 cm^3^ in apparent tumors) (21). Vargas et al. (17) showed that >50% of PCa with GS ≥ 4 + 3 were underestimated in patients with tumor volume <0.5 cm^3^. Seo et al. (22) also revealed that >50% of clinically significant PCa (PI-RADS v2 score <4) was underestimated in patients with tumor volume <1.0 cm^3^. Therefore, it is still uncertain whether the parameters of mpMRI function well in predicting GS score. It is reported that PI-RADS v2 score is associated with GS score, tumor volume, and extracapsular invasion. However, a recent study demonstrated that the positive predictive value of PI-RADS v2 = 5 for predicting LN metastasis was only 20%, while the negative predictive value was 99% (16). Therefore, PI-RADS v2 could only be used to exclude patients with an extremely low risk of LN metastasis (16). Any PI-RADS v2 score may contain more than one GS; that is, there may be overlap of various GSs between adjacent PI-RADS v2 scores (23). However, the tumor size (e.g., 1.5 cm) might become an important factor for predicting LN(+) in patients with PI-RADS v2 score of 4 or 5. In patients with GS ≥ 7, PI-RADS v2 score was 4 for small-volume PCa but could be 5 in cases with large tumors. Thus, PI-RADS v2 score performed well in predicting tumor burden to some extent, although it had poor performance in predicting GS (24). The present study revealed that PI-RADS v2 was positively correlated with preoperative clinical substage and postoperative pathological tumor burden, and the correlation was even higher in patients with higher GGG. Besides, high tumor burden indicates high risk of LN invasion, which was consistent with the previous study. Consequently, PI-RADS v2 score performed well in predicting preoperative or postoperative tumor burden in patients with GGG ≥ 3, although it was not associated with LN metastasis in the whole population. High PI-RADS v2 scores frequently suggest a high tendency toward LN metastasis.

Intraductal carcinoma of the prostate is commonly regarded as a highly aggressive malignancy, although GS cannot be applied to IDC-P (25–27). It has been suggested that whether it is IDC-P should be routinely included in pathological reports (9, 28). Hollemans et al. (8) reported that IDC-P is more prone to LN metastasis in patients with GGG = 2. The present study indicated that IDC-P was not a risk factor for LN metastasis in patients with cT2 stage PCa. This may have been because the invasive pathological features were uncommon in very-low-risk patients (GGG = 1), whereas the effect of the invasive feature of IDC-P in the risk of LN(+) was inconspicuous in high-risk PCa (GGG ≥ 3). Multicenter research with a large sample is needed to verity these results.

There were several unavoidable limitations to this study. Firstly, this was a single-center retrospective study with a small sample size. Further investigation into the relationship between primary tumor characteristics and LN metastasis in patients with organ-localized PCa is needed. Secondly, we only included patients undergoing standard LND, and patients with expanded or modified LND were not included. Therefore, it is unclear whether there is a correlation between the extent of LND and positive LN metastasis. Thirdly, biopsy cases were not included because the limited tissue could not fully reflect the pathological features of the whole glandular tissue (8). However, Antonio et al. (4) reported that the percentage of preoperative positive needles and GS of biopsy specimen >4 + 3 were predictors of LN metastasis.

## Conclusion

There was a high risk of LN metastasis in patients with cT2 PCa if they had high preoperative tPSA or high postoperative GGG. Patients with cT2 PCa and GGG ≥ 3 experienced a high risk of LN metastasis if they had high PI-RADS v2 score, high clinical stage, or high postoperative pathological tumor burden. PI-RADS v2 score performed well in predicting tumor burden in patients with GGG ≥ 3 PCa.

## Data Availability Statement

All relevant data of the study are included in the article. Further inquiries of the original data can be directed to the corresponding authors.

## Ethics Statement

The studies involving human participants were reviewed and approved by the First Affiliated Hospital of Fujian Medical University and all procedures performed in studies involving human participants were in accordance with the Ethical Standards of the Institutional Ethics Committee of First Affiliated Hospital of Fujian Medical University and with the 1964 Helsinki declaration and its later amendments or comparable Ethical Standards. Written informed consent for participation was not required for this study in accordance with the National Legislation and the Institutional Requirements.

## Author Contributions

Q-SZ: conceptualization. NX and Y-HC: data curation. S-HC: investigation. Z-BK: methodology. X-YX: project administration. S-HC and X-DL: resources. Y-PW: software. YW and J-BH: supervision. YW: validation. NX, Z-BK, Y-HC, and Y-PW: writing – original draft. X-DL and X-YX: writing – review and editing. All authors contributed to the article and approved the submitted version.

## Conflict of Interest

The authors declare that the research was conducted in the absence of any commercial or financial relationships that could be construed as a potential conflict of interest.

## References

[1] Kryvenko ON Gupta NS Virani N Schultz D Gomez J Amin A Gleason score 7 adenocarcinoma of the prostate with lymph node metastases: analysis of 184 radical prostatectomy specimens. *Arch Pathol Lab Med*. (2013) 137:610–7. 10.5858/arpa.2012-0128-OA 23627451

[2] Diolombi ML Epstein JI. Metastatic potential to regional lymph nodes with Gleason score </=7, including tertiary pattern 5, at radical prostatectomy. *BJU Int*. (2017) 119:872–8. 10.1111/bju.13623 27496532

[3] Moris L Van den Broeck T Tosco L Van Baelen A Gontero P Karnes RJ Impact of lymph node burden on survival of high-risk prostate cancer patients following radical prostatectomy and pelvic lymph node dissection. *Front Surg*. (2016) 3:65. 10.3389/fsurg.2016.00065 28018903 PMC5159485

[4] Porcaro AB de Luyk N Corsi P Sebben M Tafuri A Mattevi D Clinical factors predicting and stratifying the risk of lymph node invasion in localized prostate cancer. *Urol Int*. (2017) 99:207–14. 10.1159/000458763 28245480

[5] Schiavina R Borghesi M Brunocilla E Manferrari F Fiorentino M Vagnoni V Differing risk of cancer death among patients with lymph node metastasis after radical prostatectomy and pelvic lymph node dissection: identification of risk categories according to number of positive nodes and Gleason score. *BJU Int*. (2013) 111:1237–44. 10.1111/j.1464-410X.2012.11602.x 23331345

[6] Kim DK Koo KC Abdel Raheem A Kim KH Chung BH Choi YD Single positive lymph node prostate cancer can be treated surgically without recurrence. *PLoS One*. (2016) 11:e0152391. 10.1371/journal.pone.0152391 27031340 PMC4816527

[7] Feifer AH Elkin EB Lowrance WT Denton B Jacks L Yee DS Temporal trends and predictors of pelvic lymph node dissection in open or minimally invasive radical prostatectomy. *Cancer*. (2011) 117:3933–42. 10.1002/cncr.25981 21412757 PMC3136649

[8] Hollemans E Verhoef EI Bangma CH Rietbergen J Helleman J Roobol MJ Large cribriform growth pattern identifies ISUP grade 2 prostate cancer at high risk for recurrence and metastasis. *Mod Pathol*. (2019) 32:139–46. 10.1038/s41379-018-0157-9 30349027 PMC6300553

[9] Epstein JI Egevad L Amin MB Delahunt B Srigley JR Humphrey PA. The 2014 international society of urological pathology (ISUP) consensus conference on gleason grading of prostatic carcinoma: definition of grading patterns and proposal for a new grading system. *Am J Surg Pathol*. (2016) 40:244–52. 10.1097/pas.0000000000000530 26492179

[10] Paner GP Stadler WM Hansel DE Montironi R Lin DW Amin MB. Updates in the eighth edition of the tumor-node-metastasis staging classification for urologic cancers. *Eur Urol*. (2018) 73:560–9. 10.1016/j.eururo.2017.12.018 29325693

[11] Ross HM Kryvenko ON Cowan JE Simko JP Wheeler TM Epstein JI. Do adenocarcinomas of the prostate with Gleason score (GS) </=6 have the potential to metastasize to lymph nodes? *Am J Surg Pathol*. (2012) 36:1346–52. 10.1097/PAS.0b013e3182556dcd 22531173 PMC3421030

[12] Herlemann A Kretschmer A Buchner A Karl A Tritschler S El-Malazi L Salvage lymph node dissection after (68)Ga-PSMA or (18)F-FEC PET/CT for nodal recurrence in prostate cancer patients. *Oncotarget*. (2017) 8:84180–92. 10.18632/oncotarget.21118 29137414 PMC5663586

[13] Jilg CA Drendel V Rischke HC Beck T Vach W Schaal K Diagnostic accuracy of Ga-68-HBED-CC-PSMA-ligand-PET/CT before salvage lymph node dissection for recurrent prostate cancer. *Theranostics*. (2017) 7:1770–80. 10.7150/thno.18421 28529650 PMC5436526

[14] Lim C Flood TA Hakim SW Shabana WM Quon JS El-Khodary M Evaluation of apparent diffusion coefficient and MR volumetry as independent associative factors for extra-prostatic extension (EPE) in prostatic carcinoma. *J Magn Reson Imaging*. (2016) 43:726–36. 10.1002/jmri.25033 26303719

[15] Park JJ Kim CK Park SY Park BK Lee HM Cho SW. Prostate cancer: role of pretreatment multiparametric 3-T MRI in predicting biochemical recurrence after radical prostatectomy. *AJR Am J Roentgenol*. (2014) 202:W459–65. 10.2214/ajr.13.11381 24758681

[16] Park SY Shin SJ Jung DC Cho NH Choi YD Rha KH PI-RADS version 2: preoperative role in the detection of normal-sized pelvic lymph node metastasis in prostate cancer. *Eur J Radiol*. (2017) 91:22–8. 10.1016/j.ejrad.2017.03.009 28629566

[17] Vargas HA Hotker AM Goldman DA Moskowitz CS Gondo T Matsumoto K Updated prostate imaging reporting and data system (PIRADS v2) recommendations for the detection of clinically significant prostate cancer using multiparametric MRI: critical evaluation using whole-mount pathology as standard of reference. *Eur Radiol*. (2016) 26:1606–12. 10.1007/s00330-015-4015-6 26396111 PMC4803633

[18] Park SY Oh YT Jung DC Cho NH Choi YD Rha KH Prediction of biochemical recurrence after radical prostatectomy with PI-RADS version 2 in prostate cancers: initial results. *Eur Radiol*. (2016) 26:2502–9. 10.1007/s00330-015-4077-5 26560721

[19] Le JD Tan N Shkolyar E Lu DY Kwan L Marks LS Multifocality and prostate cancer detection by multiparametric magnetic resonance imaging: correlation with whole-mount histopathology. *Eur Urol*. (2015) 67:569–76. 10.1016/j.eururo.2014.08.079 25257029

[20] Vargas HA Akin O Shukla-Dave A Zhang J Zakian KL Zheng J Performance characteristics of MR imaging in the evaluation of clinically low-risk prostate cancer: a prospective study. *Radiology*. (2012) 265:478–87. 10.1148/radiol.12120041 22952382 PMC3480819

[21] Riviere A Cornud F Beuvon F Sibony M Legmann P Barry Delongchamps N. [Pathological findings of visible and non-visible tumors on multiparametric magnetic resonance imaging (MRI) prior to radical prostatectomy]. *Prog Urol*. (2017) 27:536–42. 10.1016/j.purol.2017.07.004 28867582

[22] Seo JW Shin SJ Taik Oh Y Jung DC Cho NH Choi YD PI-RADS version 2: detection of clinically significant cancer in patients with biopsy gleason score 6 prostate cancer. *AJR Am J Roentgenol*. (2017) 209:W1–9. 10.2214/ajr.16.16981 28418690

[23] Park SY Jung DC Oh YT Cho NH Choi YD Rha KH Prostate cancer: PI-RADS version 2 helps preoperatively predict clinically significant cancers. *Radiology*. (2016) 280:108–16. 10.1148/radiol.16151133 26836049

[24] Park SY Cho NH Jung DC Oh YT. Prostate imaging-reporting and data system version 2: beyond prostate cancer detection. *Korean J Radiol*. (2018) 19:193–200. 10.3348/kjr.2018.19.2.193 29520176 PMC5840047

[25] Trudel D Downes MR Sykes J Kron KJ Trachtenberg J van der Kwast TH. Prognostic impact of intraductal carcinoma and large cribriform carcinoma architecture after prostatectomy in a contemporary cohort. *Eur J Cancer*. (2014) 50:1610–6. 10.1016/j.ejca.2014.03.009 24703897

[26] Kimura K Tsuzuki T Kato M Saito AM Sassa N Ishida R Prognostic value of intraductal carcinoma of the prostate in radical prostatectomy specimens. *Prostate*. (2014) 74:680–7. 10.1002/pros.22786 24481730

[27] Magers M Kunju LP Wu A. Intraductal carcinoma of the prostate: morphologic features, differential diagnoses, significance, and reporting practices. *Arch Pathol Lab Med*. (2015) 139:1234–41. 10.5858/arpa.2015-0206-RA 26414467

[28] Humphrey PA Moch H Cubilla AL Ulbright TM Reuter VE. The 2016 WHO classification of tumours of the urinary system and male genital organs-part b: prostate and bladder tumours. *Eur Urol*. (2016) 70:106–19. 10.1016/j.eururo.2016.02.028 26996659

